# Granulomatous Mastitis: Imaging of Temporal Evolution

**DOI:** 10.1155/2016/3737528

**Published:** 2016-03-08

**Authors:** Ahmed Bilal, Fahad Badar Albadar, Nauman Bashir Barlas

**Affiliations:** ^1^Department of Medical Imaging, King Khalid University Hospital, Riyadh 61421, Saudi Arabia; ^2^Northumbria HealthCare NHS Foundation Trust, Wansbeck General Hospital, Ashington NE63 9JJ, UK

## Abstract

*Aim*. To assess the temporal imaging evolution of granulomatous mastitis and to review imaging findings.* Material and Methods*. Retrospective review of imaging data of 10 patients with biopsy proven granulomatous mastitis. The patients were divided into 3 groups according to their initial imaging presentation. Temporal evolution of imaging findings was observed separately for each group. Ratios, proportions, and percentages were used for data analysis.* Results*. Upon initial presentation, 75% of women who underwent mammogram showed an area of mass like architectural distortion. 25% demonstrated focal asymmetry. Complex cystic lesion was seen 40%. Multiple abscesses with sinus tract formation tracking into surrounding tissues were seen in 2 cases. Four out of 10 patients presented as edematous changes. Three out of this group progressed to develop complex cystic lesions/abscess formation. 25% presenting with complex cystic lesions or abscess at presentation showed spontaneous resolution. The remainder needed surgical treatment. The patients with abscess formation and sinus tract formation needed surgical management.* Conclusion*. Initial imaging findings in granulomatous mastitis can be variable but the eventual course and outcome is similar in most patients with surgical management required in most cases.

## 1. Introduction

Granulomatous mastitis is a well-known but rare entity even among the breast radiologists. It is an inflammatory process of the breast that was first acknowledged by Kessler and Wolloch [1] in 1972. Pathologically, it is characterized by granulomatous inflammation of breast lobules [2]. Clinically, it is a chronic mastitis. Ladies of child-bearing age are most affected by this disease process [1].

There is a great variability in diagnostic and clinical approach to the patients of granulomatous mastitis. In this respect, a valuable attempt was made by Larsen et al. [3] in establishing a protocol for imaging and clinical follow-up. However, the radiological course of the disease process and temporal changes in imaging appearances during follow-up were not emphasized.

So far, to our knowledge, isolated radiological follow-up of granulomatous mastitis has not been previously discussed. Similarly, the role of radiology in decision about management options is still to be established. This encouraged us to retrospectively review the data of patients with biopsy proven granulomatous mastitis and try to categorize the imaging findings in subgroups. We believe that, by doing this, we can present an initial guideline to the reviewing radiologist and the clinician about what to expect in the follow-up of patients with granulomatous mastitis. We also believe that, by observing the course of changes in radiological appearances of the patients with different initial imaging features, a breast radiologist might be able to predict the radiological outcome of a particular patient.

Secondary goals of our study include revisiting the presenting radiological features of this relatively rare disease.

## 2. Material and Methods

Retrospective review of the radiological data was performed. 10 women were included in our study with pathological diagnosis of granulomatous mastitis. The most significant inclusion criterion was the availability of periodic radiological follow-up. All women older than 30 years underwent mammography followed by US. MRI was also performed in few selected cases, depending upon the clinical scenario. For patients below 30 years of age, ultrasound was the imaging modality of choice, which was performed in all ladies.

Standard mammography technique was used with routine craniocaudal (CC) and mediolateral oblique (MLO) views. Ultrasound examination was performed using a high frequency 10 to 12 MHz transducer. Doppler ultrasound was used to assess vascularity in all cases. Breast MRI was performed on 1.5 Tesla (GE scanner).

Sample for histologic diagnosis was obtained in all cases by FNA ± trucut biopsy using 14/16 gauge needle. Culture for tuberculosis was requested by the clinical team in every patient.

The data comprised a single cohort of primary study group (*n* = 10) with 3 subdivisions. No control group existed in our study. The patients were divided into 3 groups according to their initial imaging presentation. Temporal evolution of imaging findings was observed separately for each group. Cumulative observations for all patients regardless of initial presentation were also made. Ratios, proportions, and percentages were used for data observation.

## 3. General Statistics

All affected women were of reproductive age group (mean, 35.2 years; range, 24–48 years). All patients initially presented with breast mass without any previous management. 60% received empirical antibiotics on basis of initial clinical assessment.

## 4. Results

### 4.1. Radiological Features at Presentation

**Figure 1:**
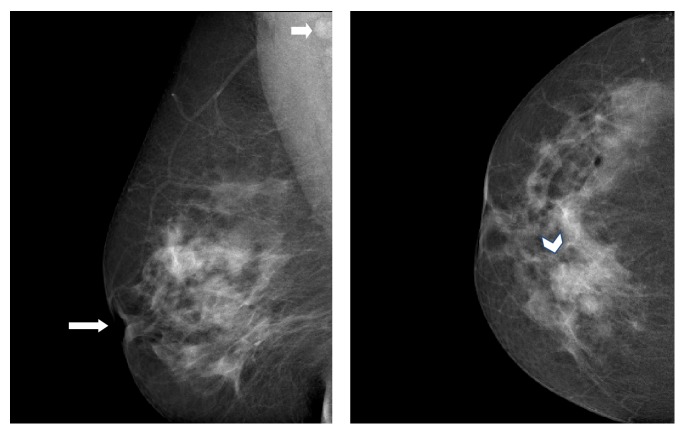
MLO and CC views of right breast. Biopsy proven granulomatous mastitis. There is an area of architectural distortion in the upper inner aspect of right breast with slightly nodular configuration and clear nipple retraction. These findings are classical for malignancy. A small rounded lymph node can be seen in axilla on MLO view.

**Figure 2:**
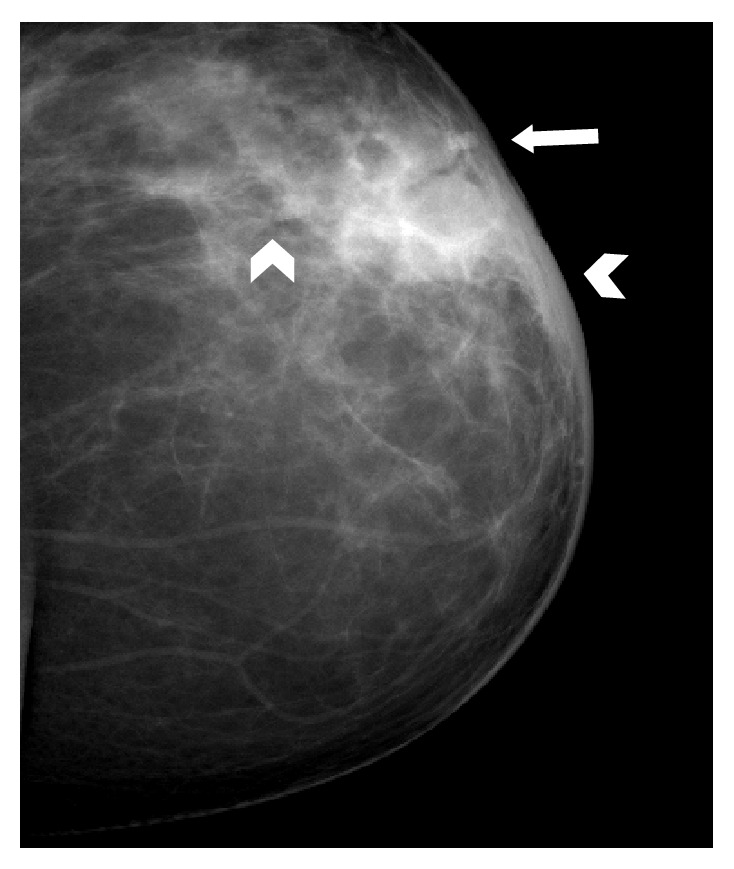
Single view of left breast. Nodular increase in retroareolar density with architectural distortion, parenchymal edema, periareolar skin thickening, and depressed nipple.

75% (*n*: 3) of women who underwent mammogram (*n* = 4) showed an area of mass like architectural distortion with parenchymal interstitial and skin edema (Figure 1). The patients with retro- and periareolar distribution showed nipple retraction as well, raising the strong possibility of malignant lesion at the time of imaging (Figure 2). The remaining 25% demonstrated a nonspecific area of focal asymmetry (*n*: 1).

**Figure 3:**
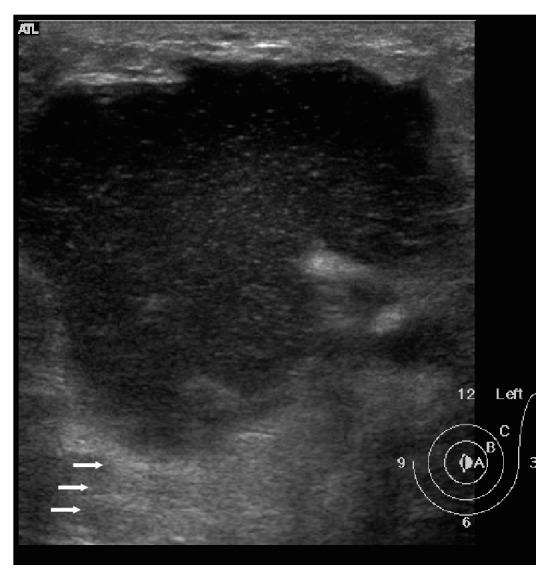
Abscess at presentation. The US image shows a relatively circumscribed hypoechoic area with internal echoes in retroareolar location. Posterior acoustic shadowing is also seen (arrows). It also demonstrated peripheral increased vascularity (not shown here), consistent with a breast abscess.

**Figure 4:**
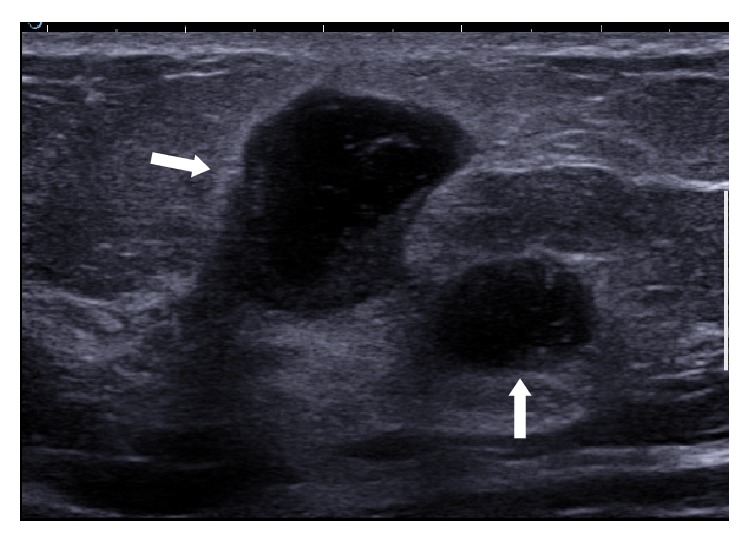
Multiple abscesses. Patient had multiple small abscesses on presentation. Two small abscesses located in close proximity are shown here.

**Figure 5:**
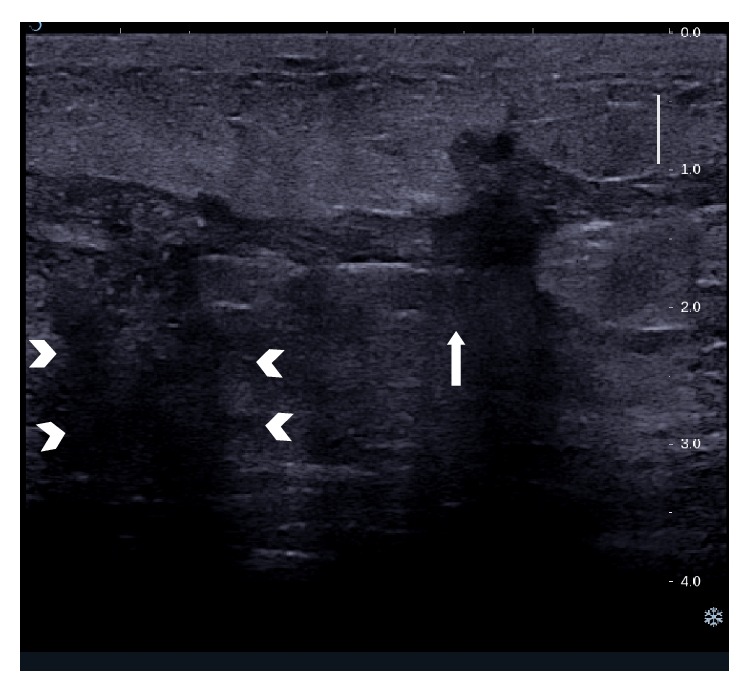
Sinus tracts. Linear sinus tracts can be seen tracking away from the abscess into surrounding tissues.

Ultrasound examination was performed in all 10 cases. It showed parenchymal edema and generalized soft tissue thickening without any discrete lesion beneath the clinically palpable abnormal area of the breast in 40% of cases (*n*: 4). Complex cystic lesion or a walled-off abscess was seen on presentation in 4 cases, 40% (Figure 3). Multiple abscesses with sinus tract formation tracking into surrounding tissues were seen in 2 cases as the presenting radiological feature (Figures 4 and 5).

**Figure 6:**
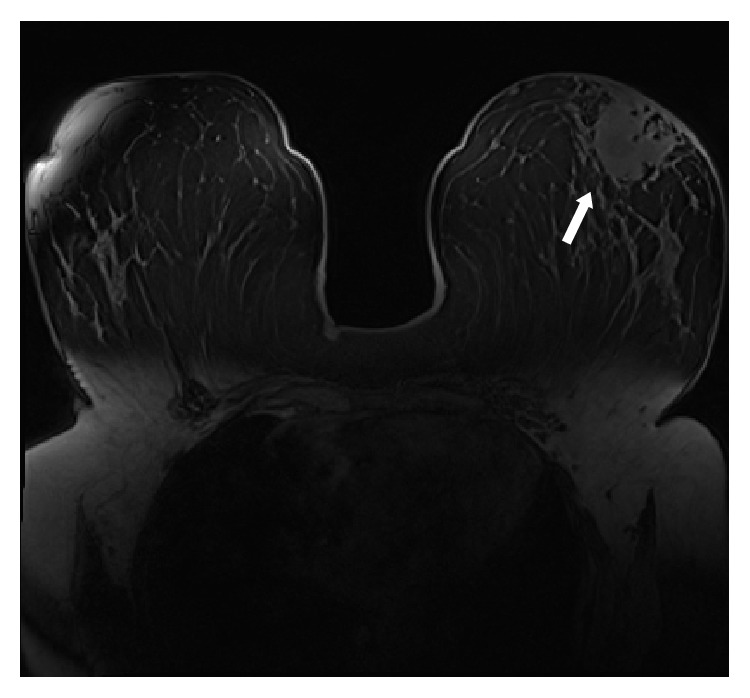
Granulomatous mastitis left breast. T1 axial postgadolinium MRI demonstrating retroareolar enhancing abnormality with periareolar skin thickening. It cannot be distinguished from a malignancy based on these imaging findings.

Four patients underwent MRI, 3 at presentation and 1 during the follow-up. MRI scans which were done at presentation confirmed the findings seen on mammogram and ultrasound. MR imaging was primarily utilized to assess the extent of disease more accurately (Figure 6).

These results are summarized in Table 1.

#### 4.1.1. Results: Follow-Up

Our primary goal was to record the radiological follow-up of these patients.

All 10 patients were treated initially by a nonsurgical approach. All patients received anti-inflammatory agents, analgesics, and antibiotics as initial treatment.

We have divided the patients into three groups according to the imaging findings on initial presentation and documented the temporal changes occurring in these patients.

**Figure 7:**
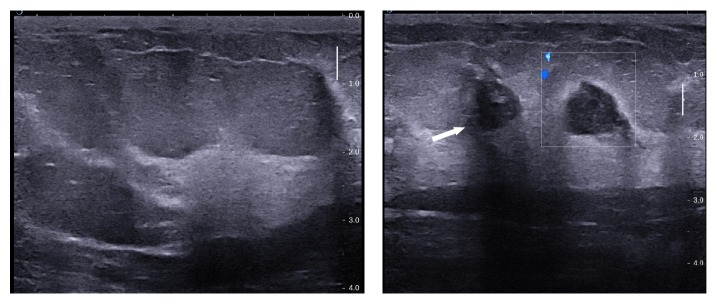
Edematous changes in the breast seen at presentation. Subsequent image shows progression of radiological features as reflected by interval development of cystic change and abscess formation (arrow).

- *Edematous Changes Only*. The disease process in patients presented as oedematous changes in 4 out of 10 patients. Three out of this group (75%) progressed to develop complex cystic lesions/abscess formation in next 6 months, despite initial treatment regime (Figure 7). Out of these 3 patients, 2 patients showed persistence of abscess for 1 year and underwent wide local excision. One of these patients with abscess formation (25%) demonstrated radiological resolution at 1 year (Table 2).
- *Complex Cystic Lesion/Abscess without Sinus Tract*. The patients presenting with complex cystic lesions or abscess at presentation partially responded to treatment with improvement in clinical features and partial resolution of radiological changes. However, complete regression without surgical intervention was seen only in 1 out of 4 patients. The remainder required surgical excision.
- *Abscess with Sinus Tract Formation*. Findings in this group comprising two patients, persisted after a period of 9 months without any significant change in imaging appearances. Eventually, surgical excision was required.

Overall, it should be noted that 7 out of 10 patients failed to demonstrate radiological resolution on the basis of medical management and had to undergo surgical treatment (70%). Overall, 9 out of 10 (90%) patients presented with or developed complex cystic changes during the course of management. Only 2 patients out of these 9 (22%) demonstrated radiological resolution without surgery.

#### 4.1.2. Postsurgical Follow-Up

**Figure 8:**
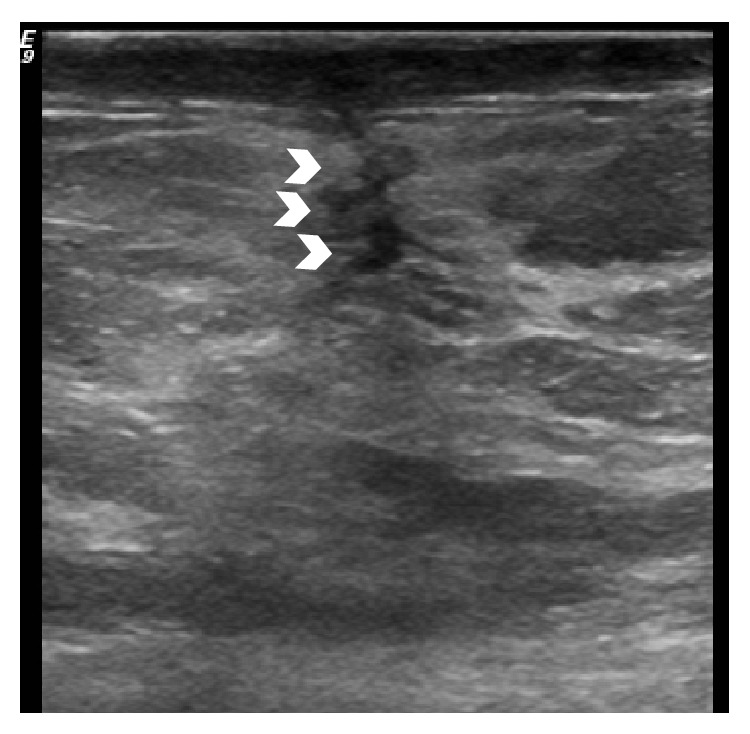
Follow-up ultrasound after surgical excision. A linear hypoechoic tract (arrowheads) can be seen at the site of surgery consistent with a surgical scar. No nodular soft tissue mass is identified to suggest residual disease.

**Figure 9:**
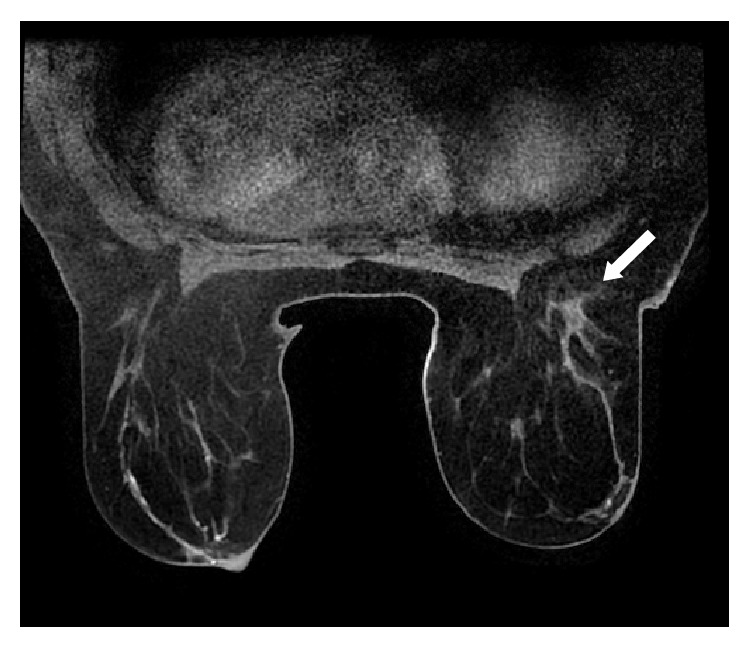
Contrast enhanced dynamic MRI of breast. The patient had surgical excision for granulomatous mastitis. Postcontrast enhancement is still seen in operative bed long time after surgery due to fibrosis in this region. It showed progressive enhancement on dynamic curve (not shown here).

Follow-up of the patients with adequate surgical resection showed a benign looking scar on ultrasound (Figure 8). However, MRI was performed in one of the postoperative cases, which demonstrated a vague area of fibrosis demonstrating mild progressive enhancement on kinetic curve (Figure 9).

## 5. Discussion

Temporal evolution of granulomatous mastitis is a less studied subject in literature. However, it is a commonly encountered problem in practice of a breast radiologist and breast surgeon to predict the imaging outcome which can potentially prevent unusual surprises for a patient and clinician. This is owing to a combination of highly variable initial presentation and no recognized association with any specific etiology. An autoimmune reaction is considered as a possible explanation [3]. Before designating the etiological factor as autoimmune, other causes such as tuberculosis, sarcoidosis, Wegener granulomatosis, fungal infections, and most importantly breast cancer should be excluded [4]. Lades of reproductive age group are primarily affected as seen in our study (mean, 35.2 years; range, 24–48 years). This is concordant with previously published results on a larger scale [3]. According to Memis et al., typical clinical presentation is painful lump associated with skin thickening and inflammatory changes [5]. Our results partially agree with this, as we also observed edema and inflammatory changes documented in all 10 cases, though in 40% of these cases it was an isolated finding, not associated with any discrete lump.

Clinically palpable breast mass was a common clinical presentation in our study being present in 6 out of 10 patients. It is notable that, among these patients, mass was tender to palpation in only 3 cases. This posed further diagnostic difficulty as the absence of pain raised the suspicion for breast cancer. More importantly, 2 patients demonstrated nipple retraction which itself created a strong suspicion for a malignant mass.

Mammographic findings for granulomatous mastitis are quite variable as described previously in literature by Han et al. [6] and Yilmaz et al. [7]. In these studies, focal asymmetry and multiple breast masses have been reported. Lee et al. [8] described an irregular mass as the most common feature. In our cases, it was reported as architectural distortion and focal asymmetry in all cases. No discrete mass was identified on mammogram. Ultrasound findings at variable time course consisted of complex cystic lesion/abscess formation with or without sinus tract formation. Edema only was seen in 40% of cases, 1 presenting with hypoechoic tracts tracking in between the breast parenchyma. Abscess formation and sinus tracts at presentation were seen in 20% of cases. Lee et al. [8] also obtained similar results.

Our primary aim was to study the temporal evolution of breast changes in granulomatous mastitis. The mildest presentation was the breast edema which was seen in 40% of cases; ultrasound demonstrated diffuse oedematous changes without any discrete changes. It was seen that 75% of these cases (*n*: 3) progressed to radiological abscess formation eventually over a period of 6 months. This was characterized by a thick walled collection with internal debris or septations and increased mural and perimural vascularity (Figure 7). These observations direct us to think that even if edema is the only radiological feature, there is a high likelihood of progression towards cyst and abscess formation in subsequent imaging.

Second group including 40% patients presented at the stage of complex cyst/abscess formation. Even at the end of the 6-month interval, only one of these patients showed resolution. Persistent imaging features were seen with no obvious change. We think that this finding would prompt the surgeons for an early surgical intervention in patients who present with abscess at initial radiological examination.

The patients presenting with multiple sinuses and tracts associated with abscess also demonstrated no significant change over a period of 9 months. Eventual management was surgical excision.

Previously, variable attempts have been made in establishment of protocols for treatment of granulomatous mastitis [9, 10]. In our view, the most elaborate and comprehensive effort has been made by Larsen et al. [3]. This was a large study group; however, it did not emphasize on the temporal evolution of imaging findings. Larsen et al. [3] discussed incision and drainage as a management option and have concluded that wide local excision is more beneficial. In our study, the management protocol was quite straightforward. All the patients received antibiotics with partial improvement seen in 30% of cases with complete resolution documented in only 2 cases without surgical treatment. Wide local excision was employed as the only surgical management option. Follow-up of these patients showed a benign looking scar on ultrasound and MRI (Figures 8 and 9).

**Figure 10:**
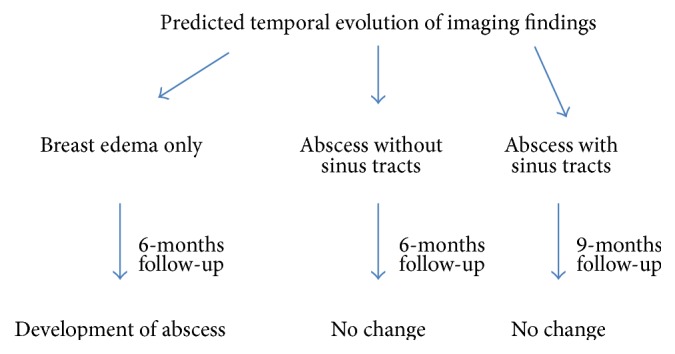
Flow chart representing the predicted temporal course of 3 subgroups.

**Figure 11:**
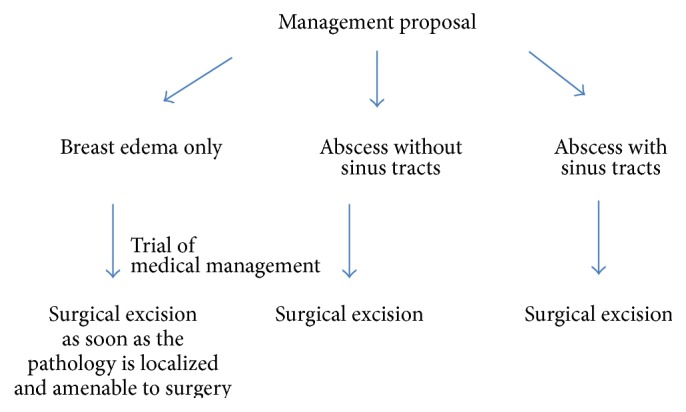
Flow chart demonstrating the management proposal of 3 subgroups.

Overall, on the basis of our results, we believe that regardless of initial presentation, high percentage of patients (70% in our case) would develop complex cystic changes in breast during the course of disease process. The likelihood of resolution without surgical management is expected to be low (22% in our case). We can infer multiple conclusions from these observations which can guide the management. First, despite the variability in initial presentation, the majority of patients tend to end up with an abscess formation. Secondly, there is a low percentage of patients which demonstrated response to medical management which implies that surgical management might be considered as a top management option. Thirdly, the patients with abscess at presentation should be given the chance of early surgery as the likelihood of improvement by medical management is low. Fourth, some patients present with interstitial edema only and it is quite understandable that surgical excision is difficult at this point due to poor localization. However, the radiologists and surgeons should not be surprised if it eventually evolves into an abscess. These patients should be cautiously and closely followed up and we suggest that, as soon as the abnormality is relatively localized, a surgical excision might be attempted. We have summarized these results and proposals in simplified flow charts (Figures 10 and 11).

We also strongly support the argument by Larsen et al. [3] based on our results and neat postoperative radiological appearances; however, we add that surgical intervention should be employed as early as possible.

Our study has several strengths and limitations. The number of patients is small in our study, the reason being that we could include only those patients who had undergone a regular imaging follow-up. Given this, we are only able to make observations with no statistical significance. This was a retrospective study with a potential for prospective design.

The greatest strength of our study is the novelty; although diagnostic and management aspects of granulomatous mastitis have been attempted before [3, 11, 12], however, to our knowledge, the temporal evolution of imaging findings has not been studied before. Although our study may not be used for drawing robust conclusions, it certainly can serve as an initial guideline to breast radiologists and breast surgeons on what to expect in imaging follow-up of these patients. Similarly, a prospective study with a larger group is also a logical next step. Another aspect of our study is that we have inferred that surgical option might as well be the earliest option in management of these patients. We believe that based on our study if a prospective group is studied to the extent of statistical significance, we might be able to altogether eliminate the option of prolonged medical management in these patients.

## 6. Conclusion

Imaging findings of granulomatous mastitis can be deceptive for a malignant process. Although initial radiological presentation is variable, there is always a strong likelihood of developing breast abscess and sinus tracts in the absence of early surgical intervention. Spontaneous resolution is also less likely. Knowledge of its temporal course of evolution in imaging is essential to avoid clinical and radiological surprises.

## Figures and Tables

**Table 1:** Imaging appearances of granulomatous mastitis on presentation.

Modality	*n* (%)
Mammogram	*n*: 4
Architectural distortion with interstitial and skin edema	*n*: 3 (75%)
Focal asymmetry	*n*: 1 (25%)

Ultrasound	*n*: 10
Parenchymal edema and soft tissue thickening	*n*: 4 (40%)
Complex cystic lesion or a walled-off abscess	*n*: 4 (40%)
Multiple abscesses with sinus tract formation	*n*: 2 (20%)

MRI	*n*: 4
Architectural distortion	*n*: 1 (25%)
Suspicious mass	*n*: 1 (25%)
Abscesses	*n*: 2 (50%)

Note: MRI findings were compatible with primary imaging modality (MG, US).

**Table 2:** Follow-up of imaging findings.

Initial course of disease	% of total	Resolution in 1 year without surgery	Need for surgical excision
Progression as complex cyst formation/abscess	75%	33%	66%
Regression	25%	100%	0%
